# Lipofuscin-mediated photodynamic stress induces adverse changes in nanomechanical properties of retinal pigment epithelium cells

**DOI:** 10.1038/s41598-018-36322-2

**Published:** 2018-12-18

**Authors:** Anna Wiktor, Michal Sarna, Dawid Wnuk, Tadeusz Sarna

**Affiliations:** 1 Department of Biophysics, Faculty of Biochemistry, Biophysics and Biotechnology, Jagiellonian University, Gronostajowa 7, 30-387, Krakow, Poland; 2 Department of Cell Biology, Faculty of Biochemistry, Biophysics and Biotechnology, Jagiellonian University, Gronostajowa 7, 30-387, Krakow, Poland

## Abstract

Retinal pigment epithelium (RPE) is an important part of the blood-retina barrier (BRB) that separates the retina from the choroid. Although melanin granules contribute to the mechanical stability of the BRB complex, it is unknown if the age pigment lipofuscin affects mechanical properties of the tissue. To address this issue the effect of sub-lethal photic stress mediated by phagocytized lipofuscin granules, isolated from RPE of human donors, on morphology and mechanical properties of ARPE-19 cells was investigated. Nanomechanical analysis using atomic force spectroscopy revealed that irradiation of cells containing lipofuscin granules with blue light induced significant softening of the cells, which was accompanied by substantial reorganization of the cell cytoskeleton due to peroxidation of cellular proteins. Our results indicate that lipofuscin-mediated photic stress can cause significant modification of the RPE cells with the potential to disturb biological function of the BRB complex.

## Introduction

Retinal pigment epithelium (RPE), a single layer of cells, located in the outermost part of the retina, plays a key role in metabolic support of the adjacent photoreceptor cells and is involved in biological renewal of photoreceptor outer segment membranes^1^. Being exposed to high oxygen tension and intense light from focal irradiation, RPE cells are at risk of oxidative stress that is aggravated by the cell photosensitizing pigments, including the age pigment lipofuscin (LF)^2^. LF accumulates in the human RPE with senescence and by the age of 40 approximately 8% of the cytoplasmic volume of macular RPE cells is occupied by lipofuscin granules^3^, whereas at the 8^th^ decade of life lipofuscin content reaches 19% of the cytoplasmic volume^4–6^.

In the RPE, LF is present in the form of distinct fluorescent granules, approximately 1 micron in diameter, containing a conglomerate of covalently cross-linked proteins (30–60%), complex lipid material and retinoid-derived chromophores^7^. In model systems, isolated lipofuscin granules showed substantial photoreactivity generating, upon excitation with blue light, singlet oxygen, superoxide anion and hydrogen peroxide, and inducing peroxidation of unsaturated lipids^8–10^. It has been postulated that phototoxic reactions, mediated by lipofuscin, can be a major contributor to chronic oxidative stress in the human RPE^5,11–13^. It can be argued that reactive oxygen species (ROS), photogenerated by lipofuscin, particularly in the aging RPE, may lead to oxidative stress and contribute to impairment of normal functions of this important tissue. One of such RPE functions is its contribution to the blood-retina barrier (BRB) that separates the retina from the choroid^14^. The breakdown of the BRB has severe consequences for proper functions of the posterior segments of the eye and occurs in several pathological conditions such as mechanical disruption, hydrostatic factors, metabolic diseases, inflammation and age-related macular degeneration^15–17^. Recently, we have shown that melanin granules, present in the RPE cells, are responsible for the exceptional stiffness and rigidity of the BRB complex^18^. However, it remains unclear if lipofuscin, the other prominent pigment of the human RPE, has any impact on the mechanical properties of RPE cells. Importantly, mechanical properties of lipofuscin granules also remain unknown.

In this study, we analyzed the effects of lipofuscin-mediated oxidative stress on the elasticity of RPE cells and their cytoskeleton organization. We also examined if the extent of cellular changes, accompanying lipofuscin-mediated photic stress, depended on age of the human donors. Changes in the cellular scaffolding – the cytoskeleton of human RPE cells can be viewed as one of the most sensitive indicators of sub-lethal oxidative modifications, accompanying chronic phototoxicity. Such changes were analyzed by laser scanning confocal microscopy (LSCM) after staining selected cytoskeleton structures, and by atomic force microscopy and spectroscopy (AFM/S). To evaluate oxidizing capabilities of the age pigment, photoperoxidation of proteins in ARPE-19 cells containing phagocytized lipofuscin granules was determined employing the sensitive fluorescent probe coumarin boronic acid (CBA).

## Results

In this study, we analyzed responses of cultured ARPE-19 cells, subjected to sub-lethal or weakly lethal photic stress, after re-pigmentation with RPE lipofuscin granules isolated from human donors of different age. 3D structure illumination microscopy revealed that LF granules were distributed all over the cells and occupied the entire volume of the cytoplasm (Supplementary Fig. S1). This confirms that the model used in our study mimics well the spatial distribution of lipofuscin in RPE tissue^19^. Initial experiments were performed to examine if LF granules, at the concentration used, were cytotoxic in darkness, and if irradiation alone induced any cell killing. The data clearly show that MTT-determined cell survival did not differ with culture time (Supplementary Fig. S2A). There was no difference in cell survival between ARPE-19 cells fed lipofuscin granules isolated from younger donors (LF_18–29) and older donors (LF_50–59). Thus, under the experimental conditions used, phagocytized LF granules, regardless the age of donors, did not exhibit any dark cytotoxicity detectable by the employed cell survival assay. As expected, irradiation of control cells, without lipofuscin, with blue light had no effect on cell survival (Supplementary Fig. S2B). Only cells preloaded with LF granules and irradiated with blue light for 2 hrs, exhibited reduced survival, with the effect being more prominent for cells containing granules from older donors (LF_50–59). Thus, while the survival of irradiated cells containing LF_18–29 was reduced by less than 10% the survival of cells containing LF_50–59 was reduced by 20%, compared to irradiated cells without lipofuscin (Supplementary Fig. S2B).

Experiments with PI fluorescence were conducted to confirm the subtle cytotoxic effect of the photoexcited LF granules that was detected by the MTT assay. PI fluorescence of ARPE-19 cells containing phagocytized LF_18–29 or LF_50–59 and irradiated with blue light for selected time intervals, was measured 24 hrs post-irradiation (Supplementary Fig. S3). The measured fluorescence was negligible after irradiation of cells containing LF_18–29 for 30 min or 1 h 30 min. Weak lethal effect, indicated by the appearance of few red nuclei was observed in cells containing phagocytized LF_18–29 and irradiated for 2 hrs. On the other hand, cells containing phagocytized LF_50–59, developed detectable PI fluorescence, even after 30 min of irradiation. Fluorescent microscopy of cells containing phagocytized LF_50–59 and irradiated for 2 hrs showed significant number of PI-positive nuclei. No PI fluorescence was observed in the case of ARPE-19 cells lacking LF particles both in the dark and irradiated with blue light of the same doses. Although results of the PI test are generally consistent with those of the MTT assay, a weak phototoxic effect was detected by PI fluorescence in cells containing LF_50–59 and irradiated for only 30 min. The data suggest that the PI test was more sensitive than the MTT assay for detecting weak phototoxic events mediated by lipofuscin.

**Figure 1:**
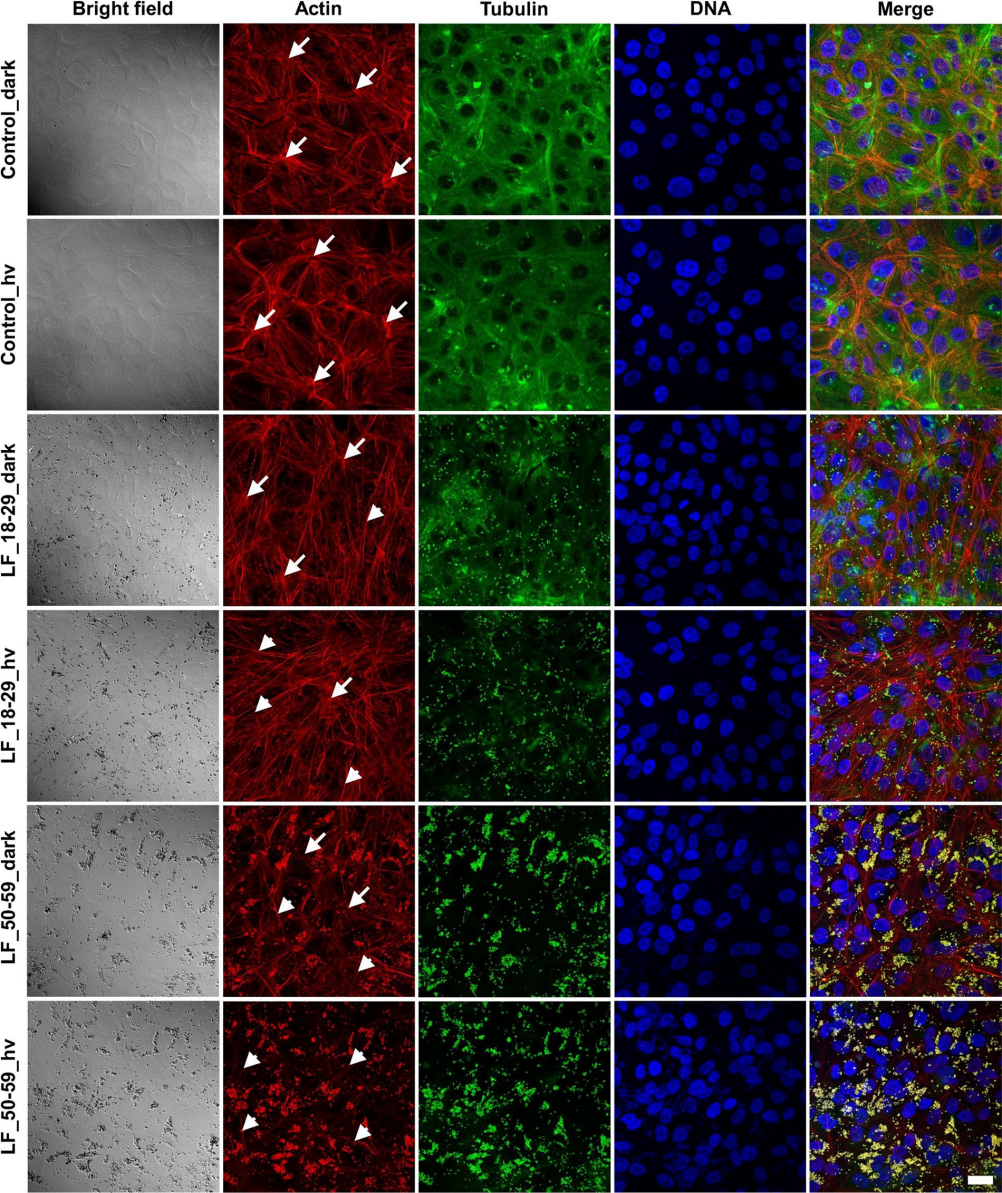
Effect of lipofuscin-mediated photic stress on the cytoskeleton of ARPE-19 cells. Scatter laser light images showing the morphology of the cells (first column from the left) followed by fluorescence images of the cells cytoskeleton (remaining columns) shown in the maximum intensity projection mode. Arrows indicate thick actin stress fibers, whereas arrow heads indicate thin actin filaments. Scale bar for all images represent 20 µm.

Laser scanning confocal microscopy analysis of cells loaded with lipofuscin and irradiated for 2 hrs with blue light were performed 24 hrs post-irradiation. Representative fluorescence images of cell cytoskeleton (actin organization and microtubules) in cells containing phagocytized LF_18–29 or LF_50–59 and in control cells are shown in Fig. 1. These images clearly indicate that irradiation alone did not cause any modifications to the cell cytoskeleton. In control cells, actin was mostly incorporated into thick stress fibers, whereas microtubules were abundant all over the cells. Importantly, these images show that actin organization in the studied cells resemble that of late (8 weeks) confluence^20^. On the other hand, in cells containing LF_18–29 particles some modifications of the actin cytoskeleton were apparent even in the dark, with actin stress fibers being thinner and less prominent than in control cells, without lipofuscin. Irradiation with blue light led to further modifications of the actin cytoskeleton leaving thin actin filaments. In cells preloaded with LF_50–59 granules, the observable effects were even more pronounced, and irradiation with blue light resulted in a complete disruption of the actin cytoskeleton leaving few individual filaments. Microtubules seemed to be more vulnerable to photic stress mediated by lipofuscin than the actin cytoskeleton. Thus, sub-lethal or weakly lethal photodynamic stress, mediated in ARPE-19 cells by phagocytized LF granules, was accompanied by significant modification of the cell cytoskeleton, considered to be the main contributor to the mechanical properties of the cells^21^.

**Figure 2:**
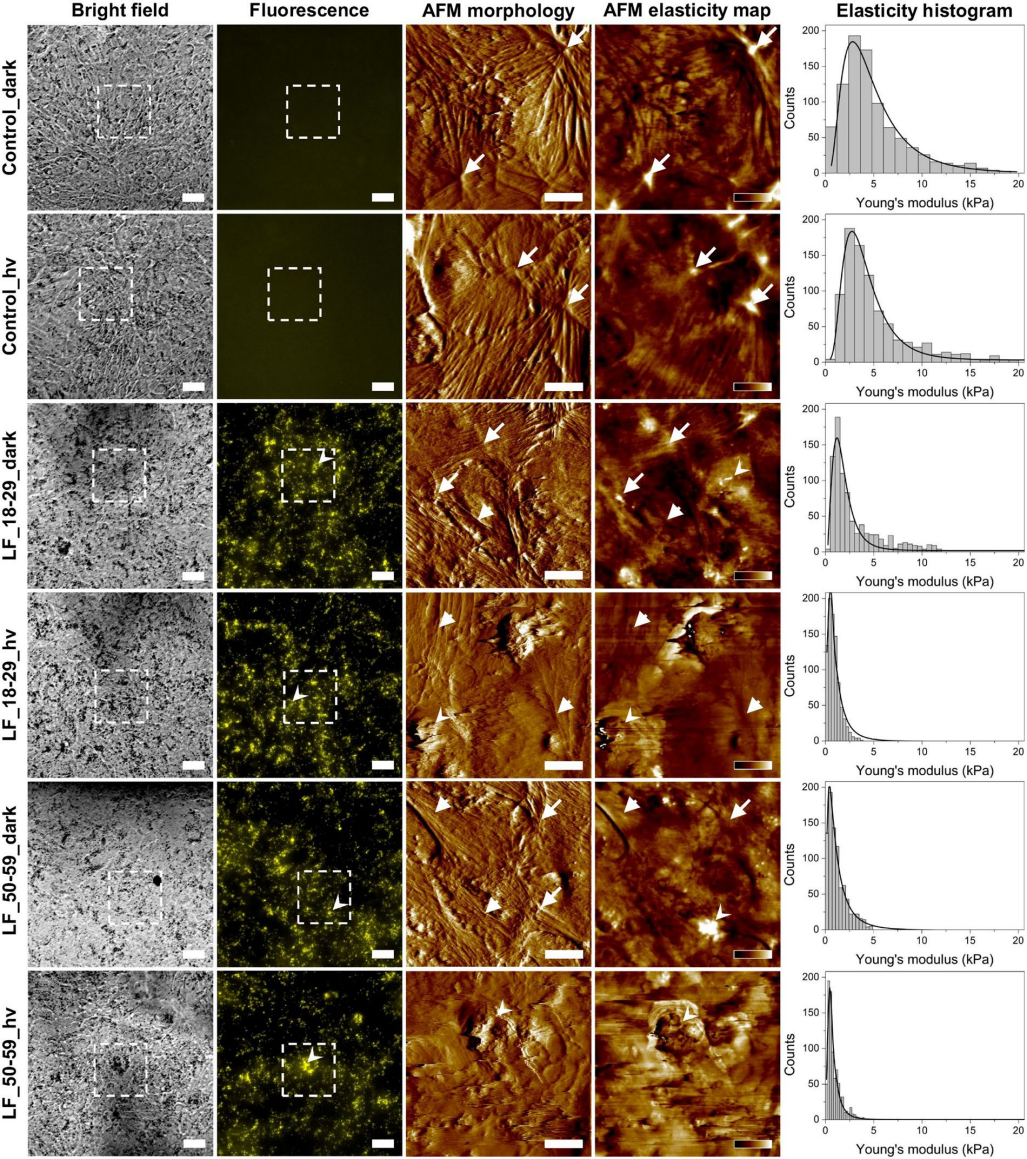
Elasticity analysis of ARPE-19 cells with phagocytized lipofuscin granules. Bright field optical microscopy images (first column) of ARPE-19 cells followed by fluorescence images of the same areas (second column). Dotted squares in the images represent scanning areas covered with AFM. Scale bars for both bright field and fluorescence represent 20 µm. In the case of fluorescence images of control cells without LF granules, camera settings were the same as for images of cells with LF particles. AFM amplitude images showing cell morphology (third column) followed by elasticity map of the Young’s modulus values (forth column). Scale bars in amplitude images represent 10 µm, whereas color bars in the elasticity maps indicate values of the Young’s modulus ranging from 0 to 20 kPa (dark-to-bright) for control_dark and control_hv cells, from 0 to 10 kPa for LF_18–29_dark cells, from 0 to 5 kPa for LF_18–29_hv and LF_50–59_hv and from 0 to 2 kPa for LF_50–59_hv cells. Arrows indicate thick actin stress fibers, arrow heads thin actin filaments, whereas sharpened arrow heads LF particles. Histograms of the Young’s modulus values (last column) with log-normal fit to the data.

AFM study of the elasticity of RPE cells subjected to sub-lethal photic stress mediated by lipofuscin provided the first experimental evidence that the accompanying oxidative stress affected basic physical properties of the cells (Fig. 2). The values of the Young’s modulus (mean ± s.d.) determined based on force spectroscopy measurements were as follows: 3.82 ± 0.06 kPa (control_dark), 3.67 ± 0.06 kPa (control_hv), 1.57 ± 0.03 kPa (LF_18–29_dark), 0.83 ± 0.02 kPa (LF_18–29_hv), 1.02 ± 0.03 kPa (LF_50–59_dark), 0.62 ± 0.01 kPa (LF_50–59_hv). It is apparent that cells without lipofuscin were much stiffer than cells containing lipofuscin and even more so than cells with lipofuscin, which were irradiated with blue light. Force mapping performed on the cells indicated that high values of the Young’s modulus reported for control cells were caused by the presence of thick stress fibers in these cells. On the other hand, cells with phagocytized lipofuscin and irradiated with blue light had the lowest values of the Young’s modulus, which was caused by disruption of their cytoskeleton induced by lipofuscin-mediated photic stress. Surprisingly, cells loaded with LF granules and kept in dark also displayed lower values of the Young’s modulus when compared to control cells. This could suggest some cytotoxic effect of lipofuscin even in the dark, which would be of considerable interest. To test this hypothesis we performed additional analysis of spatial organization of actin cytoskeleton in the cells. Supplementary Fig. S4 shows images of actin organization in ARPE-19 cells taken at different focusing levels. These images indicate different features formed by actin in the cells. In control cells, either kept in dark or irradiated with blue light, actin at the bottom of the cells forms thick and short stress fibers, whereas in cells loaded with LF granules and kept in dark these filaments become thinner and more elongated. After irradiation the effect is even more prominent. On the other hand, actin near the surface of the cells in the cortex is incorporated into thick bundles for all cells kept in dark, including for those fed LF granules. In the case of cells loaded with LF particles and irradiated with blue light actin bundles become totally disrupted. These images indicate that phagocytosis alone causes disruption of actin organization in deeper parts of the cells. Taking into consideration that actin stress fibers have the highest effect on cell mechanics^22^, the results explain why in cells loaded with LF granules and kept in dark lower values of the Young’s modulus were observed.

**Figure 3:**
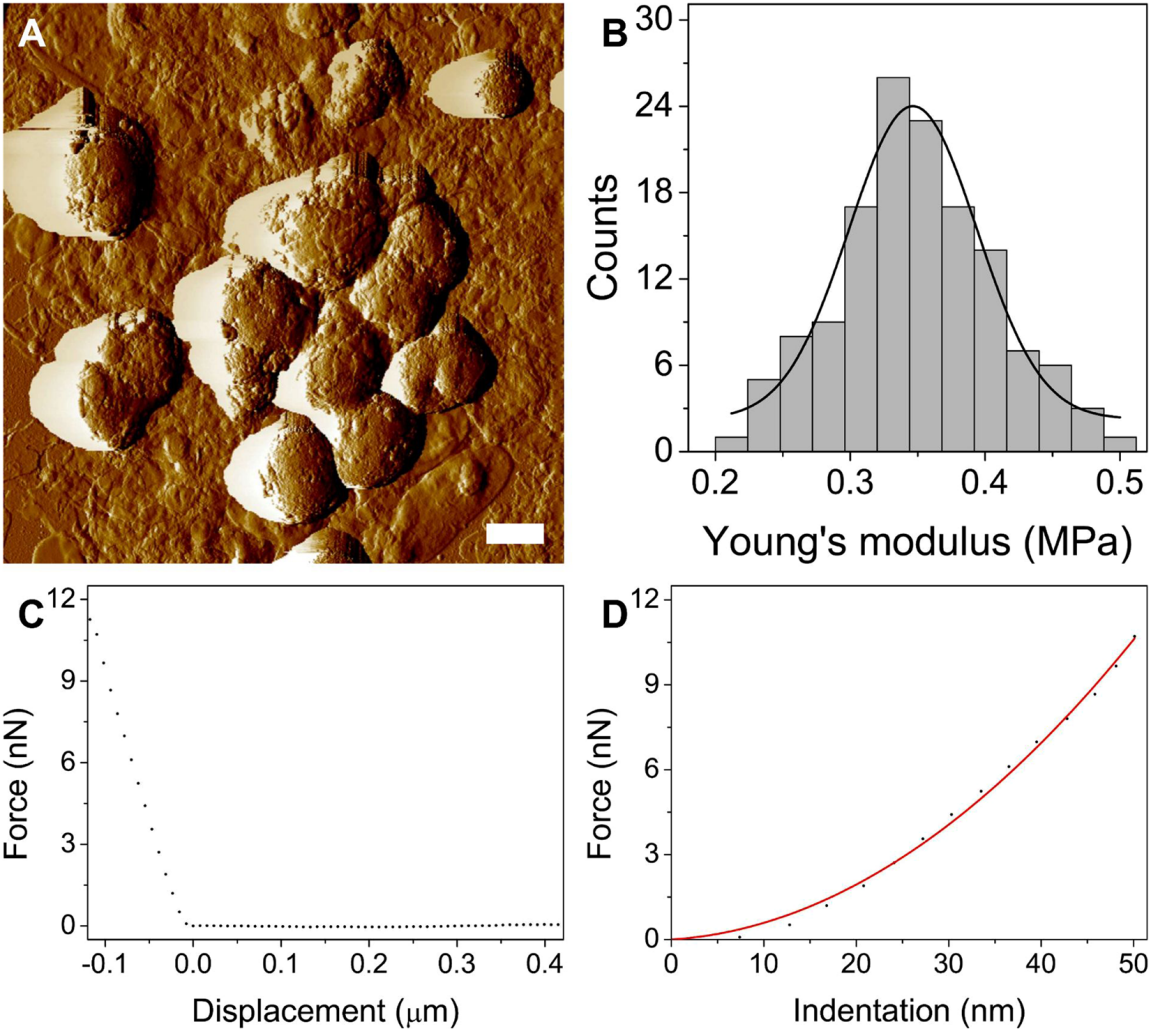
Nanomechanical properties of lipofuscin granules. (**A**) AFM amplitude image of LF particles. Scale bar represents 500 nm. (**B**) Histogram of the Young’s modulus values with Gaussian function fit to the data. (**C**) Representative force-curve obtained on a lipofuscin granule followed by force-indentation curve with the fit of the Hertz model.

To determine the nanomechanical properties of isolated lipofuscin granules, atomic force microscopy analysis was performed. Figure 3 shows representative results. Rounded morphology of LF particles (Fig. 3A) is typical for the age pigment^23^ and differs significantly from the morphology of melanosomes, which have a cigar-like shape^24^. Figure 3B shows histogram of the Young’s modulus values for LF particles examined in this work. The average value of the Young’s modulus (mean ± s.d.) was determined to be 346.49 ± 30.24 kPa, indicating that LF granules are significantly softer than human RPE melanosomes, which have an average value of the Young’s modulus on the order of megapascals^18,25^. This property of lipofuscin together with much lower number of the granules in the examined cells than that of melanosomes analyzed in our previous studies, in which the effect of melanin pigmentation on nanomechanical properties of cells was found to be significant^26–29^, indicate that the effect of LF granules on the nanomechanical properties of RPE cells is relatively minor.

**Figure 4:**
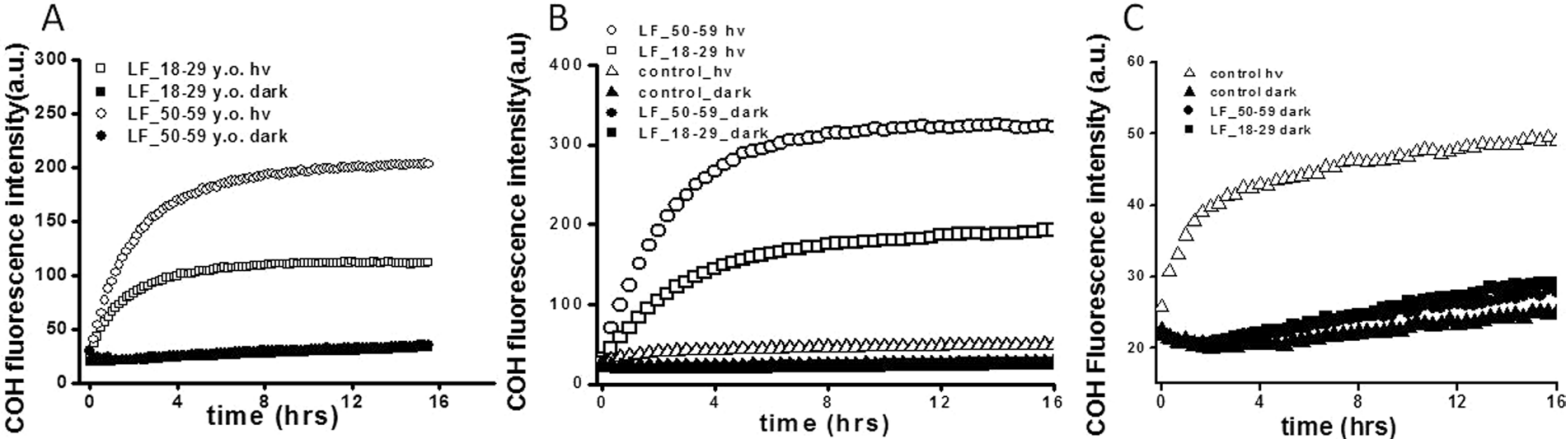
Lipofuscin-mediated oxidation of proteins in a model system and in ARPE-19 cells. Evolution of COH fluorescence in model system (**A**) containing albumin and lipofuscin from younger (LF_18–29 y.o) or older (LF_50–59 y.o), after irradiation with blue light for 30 min or kept in dark. COH fluorescence for: irradiated samples LF_18–29_hv (open squares), LF_50–59_hv (open circles) or non-irradiated samples LF_18–29_dark (solid squares), LF_50–59_dark (solid circles). Slopes and the maximum levels of the detected COH fluorescence differed significantly between irradiated lipofuscin granules versus non-irradiated (Graph Pad Prism 5 slope analysis, P < 0.0001). (**B**,**C**) Evolution of COH fluorescence in ARPE-19 cells lysates after feeding the cells with LF_18–29 y.o. or LF_50–59 y.o. granules and irradiating with blue light for 2hrs. COH fluorescence for: control, irradiated cells (open triangles), LF_18–29_hv (open squares), LF_50–59_hv (open circles) or non-irradiated control cells dark (solid triangles), LF_18–29_dark (solid squares), LF_50–59_dark (solid circles). Slopes and the maximum levels of the detected COH fluorescence differed significantly between irradiated lipofuscin granules versus non-irradiated (Graph Pad Prism 5 slope analysis, P < 0.0001).

To confirm photoreactivity of the age pigment lipofuscin, and to determine the possible mechanism of the observed cytoskeleton changes, the ability of lipofuscin to photooxidize proteins was examined (Fig. 4). First, photooxidation of albumin was determined in a model system. Measurements of the fluorescence intensity of 7-hydroxycoumarin (COH), the oxidation product of coumarin boronic acid (CBA), used as a sensitive indicator of protein hydroperoxides, revealed that irradiation of LF granules in the presence of albumin led to the protein oxidation (Fig. 4A). The observable photoperoxidation of albumin was more pronounced in samples containing LF from older donors (LF_50–59) than in samples containing LF from younger donors (LF_18–29), as evident from differences in the initial rates of the fluorescence intensity evolution determined in the examined samples. No measureable fluorescence of COH developed in dark controls with LF_18–29 and LF_50–59. Relative rates of COH fluorescence evolution in irradiated samples were as follows: 347 ± 24.6 for LF from younger donors and 589 ± 37.8 for LF from older donors. The corresponding values obtained in the dark were: 12.8 ± 0.9 for LF_18–29_dark, and 12.3 ± 1.8 for LF_50–59_dark. It is quite apparent that the rates of fluorescence evolution differed significantly between samples with irradiated LF granules and those kept in the dark.

After confirming that LF granules photoxidized albumin in a simple model system, we examined if measurable oxidation of cellular proteins could also be detected in irradiated ARPE-19 cells containing phagocytized LF granules, and, if so, whether the extent of protein oxidation depended on the age of lipofuscin donors. Representative data are also shown in Fig. 4. After irradiation of APRE-19 cells containing LF_18–29 or LF_50–59, COH fluorescence increased in the cell lysates with the effect been more prominent in samples with LF granules from older donors (Fig. 4B). No COH fluorescence was observed in lysates of non-irradiated cells and only a small increase of the fluorescence signal was detected in control irradiated cells (Fig. 4C). The initial relative rates of COH fluorescence were 45.9 ± 0.8 (control_hv), 435 ± 27.8 (LF_18–29_hv), 659 ± 43.2 (LF_50–59_hv), 4.6 ± 0.6 (control_dark), 7.8 ± 1.1 (LF_18–29_dark), and 7.1 ± 0.8 (LF_50–59_dark). The rates differed significantly between irradiated samples containing LF granules versus non-irradiated samples.

## Discussion

Studies of age-related phenomena, such as chronic oxidative stress induced in the outer retina by sub-lethal photic stress, which in the human eye may contribute to the development of age-related macular degeneration (AMD), are inherently difficult and have severe limitations. This is because there are no adequate animal models of AMD except monkeys, which for obvious reasons are not easily available for such a research. It was previously shown that phagocytosis of melanosomes or control black latex beads induced a transient upregulation of HO-1and GPx, which could modulate the susceptibility of RPE cells to photodynamic stress^30^. Therefore, the effects of lipofuscin photoactivation with blue light were examined seven days after phagocytosis of LF granules, when the cellular levels of enzymatic antioxidants returned to normal. The obtained results indicated insignificant effect of 0.5 h and 1 h irradiation of cells preloaded with LF granules on the cell survival (data not shown). Even though the killing potential of lipofuscin photoactivated with blue light has been demonstrated in cultured human cells previously^31^, in this study we aimed at examining effects of sub-lethal photic stress. We wanted to determine if blue-light irradiation of ARPE-19 cells containing phagocytized LF granules, isolated from donors of different age could modify the cell morphology and nanomechanical properties. This is an important issue considering results of our independent study, which demonstrated reversible inhibition of specific phagocytic activity of ARPE-19 cells subjected, after phagocytosis of lipofuscin granules, to sub-lethal photic stress^32^.

No measurable oxidation of cellular proteins in lysates of cells containing phagocytized lipofuscin granules and kept in darkness is somewhat puzzling in view of the observed disruption of the actin cytoskeleton and modifications of the cell elasticity in such cells. The data suggest that the mechanism of dark and light-induced effects mediated by lipofuscin granules may be different. While the phototoxicity of lipofuscin is most likely due to oxidative stress induced in the cells by photogenerated reactive oxygen species, the dark effect of lipofuscin, observed as disorganization of the actin cytoskeleton, probably does not involve significant oxidative modifications of the cytoskeleton proteins. It is most likely caused by phagocytosis, which disrupts actin stress fibers in the cells due to intracellular localization of LF granules, which was in close proximity to the stress fibers. However, we have never observed such an effect on the cytoskeleton of ARPE-19 cells loaded with melanosomes or latex beads.

Presence of LF particles, unlike presence of melanin granules, did not cause any significant stiffening of the cells. On the contrary, LF_18–29 induced a decrease in the Young’s modulus of the cells with the effect been more prominent for cells with LF_50–59. Although it cannot be ruled out that the phagocytosis itself contributed to the observed nanomechanical changes, it is striking that the effect was more pronounced in the case of LF granules from older donors. The results are consistent with the results discussed in the previous section, which demonstrated the effect of lipofuscin granules on cell morphology in the dark. Biological impact of possible dark cytotoxicity of the age pigment is unknown; however, if confirmed by independent studies, it would deserve further investigation. We have previously shown that purified human RPE lipofuscin granules, irradiated with short-wavelength visible radiation, generate singlet oxygen, superoxide anion, hydrogen peroxide and induce peroxidation of unsaturated lipids^8,10,33^.

The key result of this study is the demonstration that sub-lethal or weakly lethal photic stress, mediated by phagocytized human RPE lipofuscin granules affects nanomechanical properties of ARPE-19 cells. Oxidative stress, accompanying the photic stress, leads to oxidation of cellular proteins and disruption of the cell cytoskeleton. The changes are responsible for adverse modification of the cell elasticity. It is postulated that chronic oxidative stress in the human RPE, mediated by the age pigment lipofuscin, decreases the cell stiffness and, ultimately, may compromise the blood-retina barrier. We further postulate that such changes in the human RPE, augmented by aging, can contribute to pathogenesis of age-related macular degeneration.

## Materials

Minimum essential medium (MEM), streptomycin, penicillin, albumin, MTT, 3-(4,5- dimethylthiazol-2-yl)-2,5-diphenyltetrazolium bromide were obtained from Sigma-Aldrich Biochemie GmbH (Hamburg, Germany), and fetal bovine serum (FBS) from Gibco-Invitrogen (Auckland, New Zealand). Ethyl alcohol, dimethyl sulfoxide (DMSO), sucrose was purchased from Polskie Odczynniki Chemiczne (Gliwice, Poland). Coumarin boronic acid (CBA) was kindly provided by Dr. Radoslaw Michalski (Institute of Applied Radiation Chemistry, Technical University of Lodz, Poland).

### Methods

#### Lipofuscin isolation

Isolation of lipofuscin was performed according to the method described elsewhere^34^ and was approved by the Bioethics Committee at the Jagiellonian University (permission no. KBET/10/B/2013 obtained on January 31^st^, 2013). Briefly, human RPE cells, after isolation from donors of different age, were suspended in PBS, frozen in liquid nitrogen (LNT) and stored at LNT until further use. RPEs were collected and made available to us by the Niepubliczny Zakład Opieki Zdrowotnej FRK Homograft Sp. z o. o., Zabrze, which has all necessary permissions. RPE cells were pooled and divided into two age groups: younger (18–29 year old) and older (50–59 years old). Suspension of human RPE cells, after homogenization was diluted with PBS/EDTA solution and centrifuged for 7 min at 60 g at 4 °C (Sigma 3-16KL, Germany). Pellet was discarded and supernatant was centrifuged at 5545 g for 10 min at 4 °C (Sigma 3-16KL, Germany). The pellet obtained after second centrifugation was resuspended in 0.3 M sucrose and layered on the top of 8-step sucrose gradient (2.0 M, 1.8 M, 1.6 M, 1.55 M, 1.5 M, 1.4 M, 1.2 M and 1.0 M), then ultracentrifuged for 1 h at 103000 g at 4 °C (Beckman Coulter, Optima L-90 K, USA). Lipofuscin granules were identified as a faint orange band at the 1.0 M/1.2 M and 1.2 M/1.4 M interfaces. Lipofuscin granules, isolated from 30 eye donors of each age group, denoted as LF_18–29 and LF_50–59, were suspended in PBS and stored at −80 °C until further use. All methods were performed in accordance with the relevant guidelines and regulations.

#### Lipofuscin phagocytosis

For phagocytosis, confluent ARPE-19 cultures at 7 days after plating were fed lipofuscin granules of the selected age group. LF were delivered to cultures in complete culture medium MEM with 10% FBS. Cells were fed LF granules at a concentration of 3 × 10^7^ per ml three times every third day. Three wells per each experimental condition were used and four independent experiments were performed.

#### Cell cultures and determination of cell survival

APRE-19 cells were cultured according to the method described elsewhere^35,36^, at 37 °C in 5% CO_2_ using Minimal Essential Medium supplemented with 10% FBS. Every 7 days cells were passaged. Most of the experiments were carried out using cells in early confluence. Depending on the specific experiments, cells were seeded on plastic dishes or multiwell plates. Survival of cells subjected to lipofuscin-dependent photic stress, compared to control cells, was determined by standard techniques: the uptake by nonviable cells of fluorescent propidium iodide and reduction of 3-[4,5-dimethylthiazo-2-yl]-2,5-diphenyl tetrazolium (MTT) to distinct blue form by viable cells. The corresponding absorbance or fluorescence changes were monitored using a plate reader (GENios Plus Tecan, GmbH, Austria)^37^.

#### Blue light irradiation

ARPE-19 cells containing phagocytized lipofuscin granules were irradiated in HBSS buffer, at room temperature, for different time intervals up to 2 hrs employing a dedicated Fully Reflective Solar Simulator-SSUV1.6KW (Sciencetech Inc., Ontario, Canada) equipped with the supplied band-pass filter (400–700 nm) and blue film filter (390–510 nm) (Lee Filters, Central Way Walworth Industrial Estate Andover, Hampshire, England). The fluence rate, at the sample position, in the spectral region 402–510 nm, was 11.4 mW/cm^2^. Control experiments to monitor temperature during cell irradiation demonstrated that the temperature of the irradiated samples was never higher than 35 °C.

#### Atomic force microscopy

Atomic force microscopy analysis was conducted using Bruker’s BioScope Catalyst AFM coupled with an Axio Observer Z1 (Carl Zeiss MicroImaging GmbH, Goettingen, Germany) inverted optical microscope. Measurements of cells were performed in culture medium at 37 °C, whereas lipofuscin granules were analyzed in air at room temperature. Mechanical analysis of cells and LF particles was made in force spectroscopy mode. For cells, optical microscopy image was used to position the AFM probe on top of the cells. Once a region of interest was selected, force curves from a grid of 6 × 6 points were collected. 25 cells for each condition were analyzed. In the case of LF particles, AFM image was first made to precisely localize the granule and then force-curves were collected from selected points on the granule. Mechanical analyses of cells were made with soft cantilevers with a nominal tip radius of 20 nm and spring constant of 0.01 N/m, whereas LF particles were analyzed with stiffer cantilevers with a nominal tip radius of 8 nm and spring constant of 1.8 N/m. In the case of cells, in addition to traditional force spectroscopy measurements, force mapping was performed using the PeakForce Tapping mode with the PeakForce Capture turned on. This resulted in acquiring a force curve in each pixel of an image. For PeakForce imaging a relatively soft cantilevers with a nominal tip radius of 20 nm and spring constant of 0.68 N/m was chosen. For high precision of the mechanical analyses spring constants of the used cantilevers were routinely determined based on the thermal tune procedure as described elsewhere^38^. Analysis of force curves, obtained in force spectroscopy mode and reconstruction of force maps from force curves obtained during PeakForce imaging. was made using the AtomicJ software^39^. In brief, force-displacement curves were converted into force-indentation curves and fitted with an appropriate model. In the case of cells, where indentation was large, the Sneddon model was used, whereas in the case of LF particles in which low indentation was obtained, the Hertz model was employed. Detailed description of the mechanical analysis used in this work can be found elsewhere^40^.

#### Immunofluorescence analysis

Analysis of the cells cytoskeleton was made on samples fixed with 3.7% formaldehyde, permeabilised with 0.1% Triton X-100 and blocked with 1% bovine serum albumin at room temperature. Control cells and cells containing phagocytized lipofuscin granules, irradiated with blue light for 2hrs, were immunostained with mouse monoclonal anti-human α-tubulin IgG (Sigma-Aldrich) and Alexa Fluor 488-conjugated goat anti-mouse IgG (A110011, Life Technologies), and counterstained with Alexa Fluor 568-phalloidin (Life Technologies) and Hoechst 33342 dye for DNA staining (Sigma-Aldrich). Images were obtained using a scanning laser confocal microscope (LSM 880 from Zeiss).

#### Determination of protein peroxidation by CBA assay

To monitor oxidation of albumin in a model system and proteins in ARPE-19 cells, coumarin boronic acid (CBA) was employed as a sensitive indicator. In the presence of hydroperoxides, the non-fluorescent CBA is converted into highly fluorescent 7-hydroxycoumarin (COH)^41,42^. Fluorescence emission was measured at 465 nm after excitation at 360 nm, using the Tecan plate reader. Control or LF-loaded cells after irradiation in Hank’s Balanced Salt Solution (HBSS) were incubated for 2 min with catalase (580 U/ml). After washing with PBS, cells were scraped and centrifuge for 2 min at 60 g (Sigma, 3-16KL). Pellets were suspended in lysis buffer containing DTPA (0.1 mM) and catalase (100 U/ml), and dispersed by forcing the cells through a narrow needle. Cell lysates or albumin (500 µM) samples with LF granules were transferred into black 96-well plates and incubated with phosphate buffer (50 mM, pH 7.4), CBA (0.8 mM) and catalase (100 U/ml), and fluorescence was measured at 10 min intervals (in model system with albumin) or at 20 min intervals (in cells lysates) over 16 hrs using the Tecan plate reader. Triplicate culture wells or dishes were used for all experimental groups, respectively. The extent of protein oxidation was given as a rate of COH formation (initial increase of the fluorescent intensity normalized to time). Concentration of lipofuscin granules used in all experiments was 3 × 10^7^/ml.

### Statistical analysis

Measurements were triplicates within each series of two – four independent experiments. Statistical significance of differences in mean values was assessed using two-sample independent Student’s *t*-test at the 95% confidence level. Statistical analysis was made using Mathematica 8.0 software.

## Electronic supplementary material

Supplementary Dataset 1
