# Supplementary material for: Lipofuscin-mediated photodynamic stress induces adverse changes in nanomechanical properties of retinal pigment epithelium cells
Source: Sci Rep. 2018 Dec 18;8:17929. doi: 10.1038/s41598-018-36322-2 (PMC6298986; doi:10.1038/s41598-018-36322-2)
Supplement: Supplementary file 1 — Supplementary Dataset 1 [file 41598_2018_36322_MOESM1_ESM.pdf]

Supplementary Information For:

Lipofuscin-mediated photodynamic stress induces adverse changes in nanomechanical properties of retinal pigment epithelium cells

Anna Wiktor[1,+], Michal Sarna[1,+], Dawid Wnuk[2] and Tadeusz Sarna[1,*]

[1]Department of Biophysics, Faculty of Biochemistry, Biophysics and Biotechnology, Jagiellonian University, Gronostajowa 7, 30-387 Krakow, Poland

[2]Department of Cell Biology, Faculty of Biochemistry, Biophysics and Biotechnology, Jagiellonian University, Gronostajowa 7, 30-387 Krakow, Poland

*tadeusz.sarna@uj.edu.pl

[+]these authors contributed equally to this work

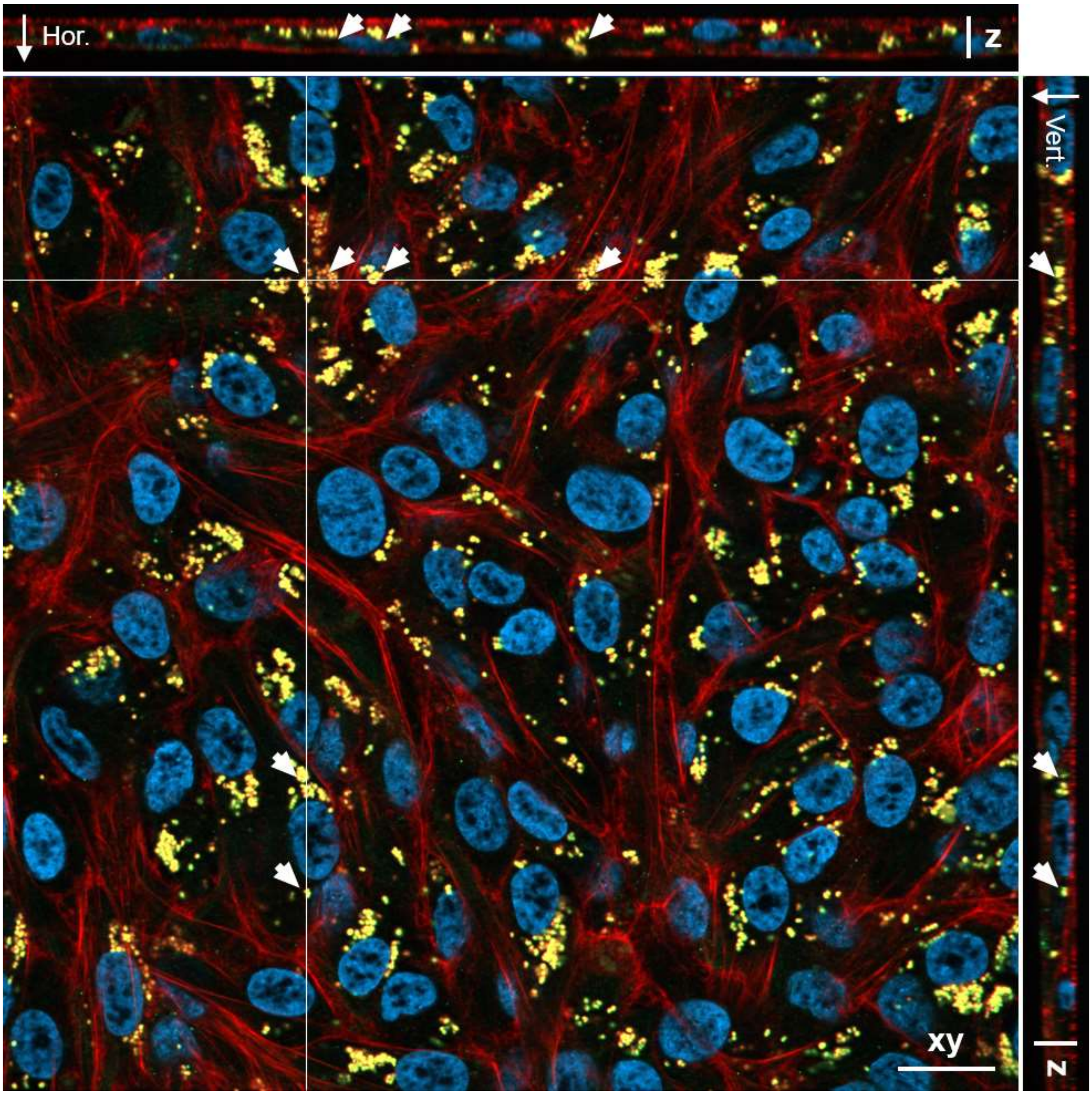

Figure S1. Spatial localization of LF granules in ARPE-19 cells. Cross sections of ARPE-19 cells loaded with LF granules. Single plane image taken at the middle of the cell monolayer and corresponding cross sections. White lines in the center image show the direction of cross sectioning: horizontal line indicates sectioning in the horizontal direction and the corresponding cross section is shown above the central image, whereas vertical line indicates sectioning in the vertical direction and the corresponding cross section is shown next to the central image. Arrows in cross sections indicate the direction towards the top of the cells away from the cell bottom.

Arrow heads indicate LF granules seen in the center image and in corresponding cross sections. Scale bars for ‘xy’ indicate 20 μm, whereas for ‘z’ 10 μm.

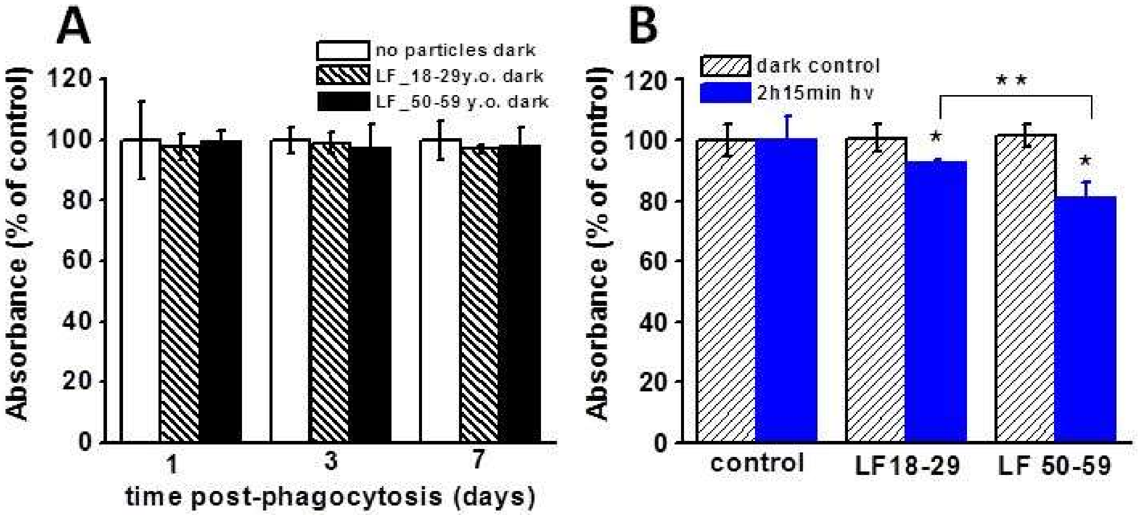

Figure S2. Effect of phagocytosis of lipofuscin granules and irradiation with blue light on survival of ARPE-19 cells. (**A**) MTT analysis of cell survival after different time post-phagocytosis. Data are the means (error bars indicate SD), from triplicate cultures, expressed in arbitrary units (a.u.) and shown as a percent of the absorbance of three replicate cultures within the 96-well plate expressed as cell survival fraction and shown as a percent of non-irradiated control cells without particles (100%), in accordance with time post-phagocytosis. (**B**) The light effect on the survival of ARPE-19 cells containing phagocytized lipofuscin granules. ARPE-19 cells with and without particles were irradiated with intense blue light for 2h (blue bars). Non-irradiated cells are expressed as black hatched bars. Data are the means (error bars indicate SD), from triplicate cultures, expressed in arbitrary units (a.u.) and shown as a percent of the absorbance of three replicate cultures within the 96-well plate expressed as cell survival fraction and shown as a percent of irradiated control cells without particles represent 100%. Four independent experiments were performed.

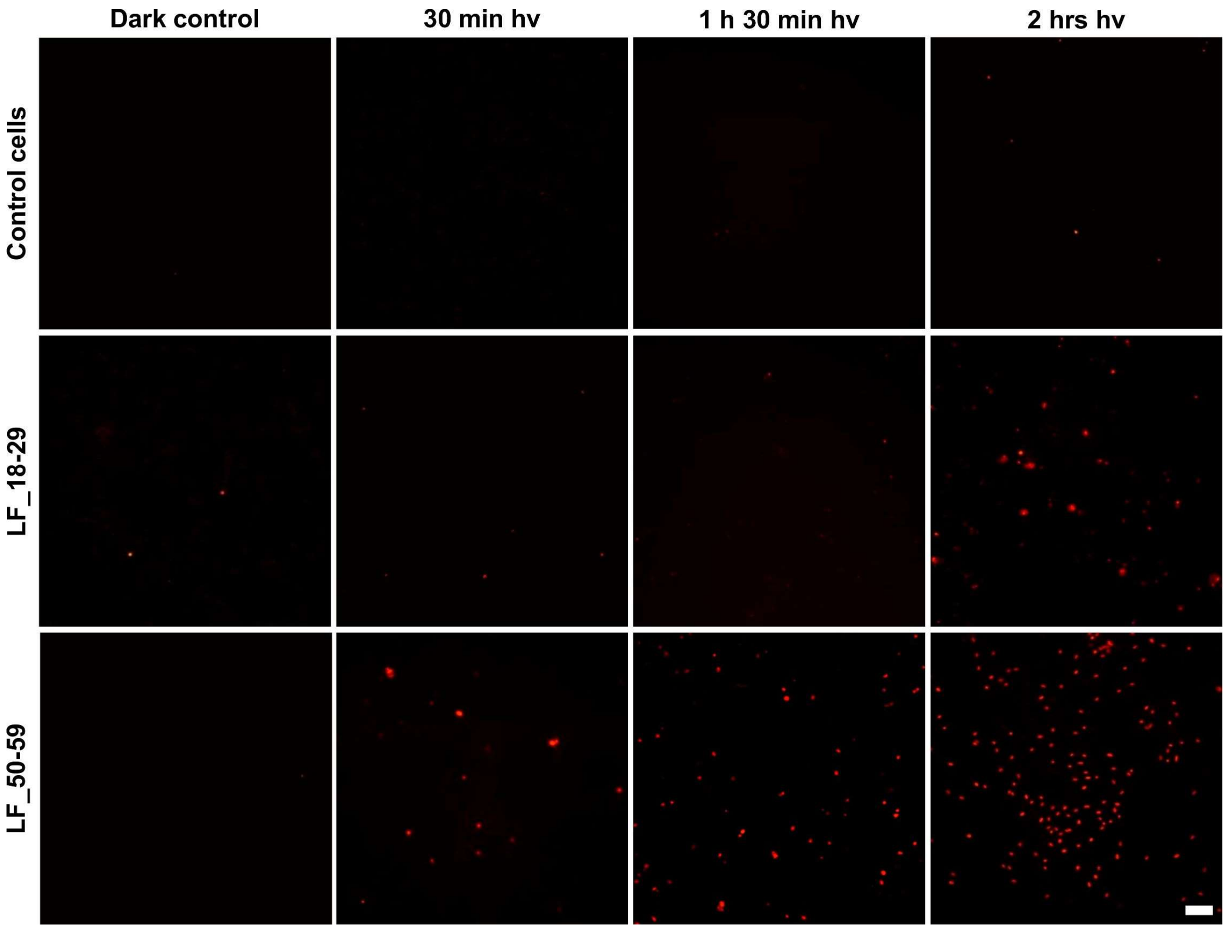

Figure S3. Propidium iodide fluorescence of ARPE-19 cells containing phagocytized lipofuscin granules and irradiated with blue light. Control ARPE-19 cells and cells containing phagocytized LF_18-29 or LF_50-59 were irradiated for selected time intervals with blue light. Panels show images of cultures at the end of the experiment (24 hr) showing PI-positive nuclei (on red). Scale bar for all images represents 100 μm.

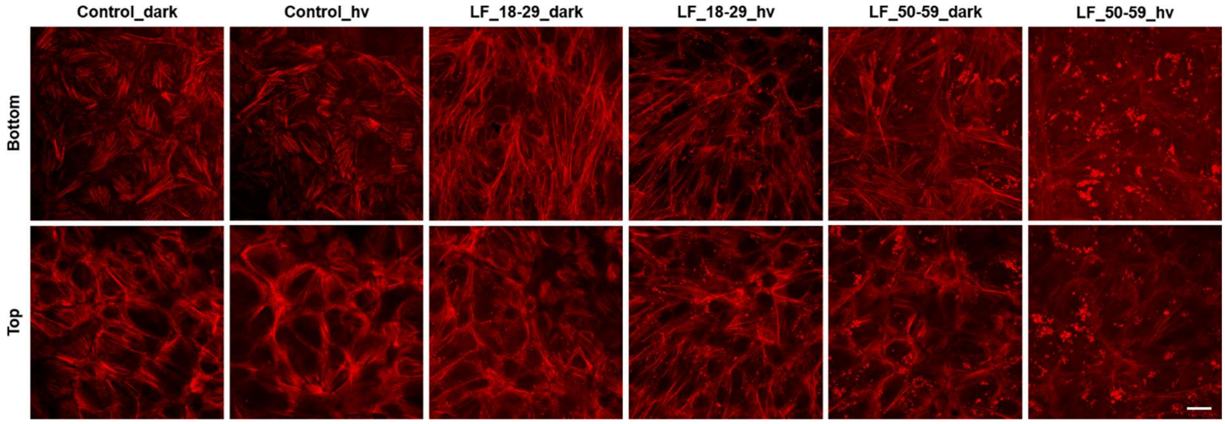

Figure S4. Spatial organization of actin cytoskeleton in ARPE-19 cells. Confocal microscopy images of actin cytoskeleton taken at two different focusing levels: at the bottom of the cells near the glass coverslip (first row), and near the cells surface (second row). Scale bar for all images indicates 20 µm.
